# Supplementary material for: Predicting Progression of Kidney Injury Based on Elastography Ultrasound and Radiomics Signatures
Source: Diagnostics (Basel). 2022 Nov 3;12(11):2678. doi: 10.3390/diagnostics12112678 (PMC9689562; doi:10.3390/diagnostics12112678)
Supplement: Supplementary file 1 [file diagnostics-12-02678-s001.zip › diagnostics-1924820-supplementary.pdf]

Supplementary Materials

Supplementary Figure S1. The flowchart of the study cohort

SWE, shear wave elastography ultrasound.

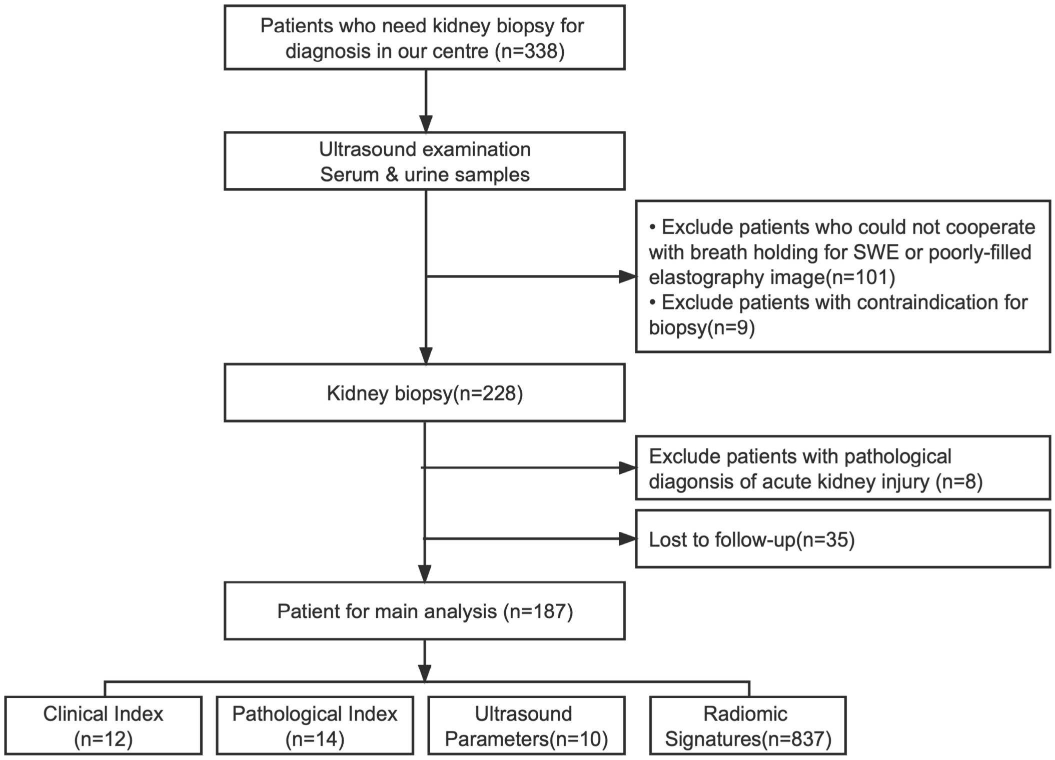

Supplementary Figure S2. Value of shear wave elastography ultrasound in the left kidney cortex, sinus, medulla grouped by BMI.

BMI, body mass index; BMI<20 kg/m^2 is defined as Below, 20-30 kg/m^2 Normal, >30 kg/m^2 Over; L_C_mean, mean SWE value of left renal cortex; L_C_median, median SWE value of left renal cortex; L_M_mean, mean SWE value of left renal medulla; L_M_median, median SWE value of left renal medulla; L_S_mean, mean SWE value of left renal sinus; L_S_median, median SWE value of left renal sinus. Kruskal-Wallis test was applied in with-group difference. A $p<0.05$ was considered significant.

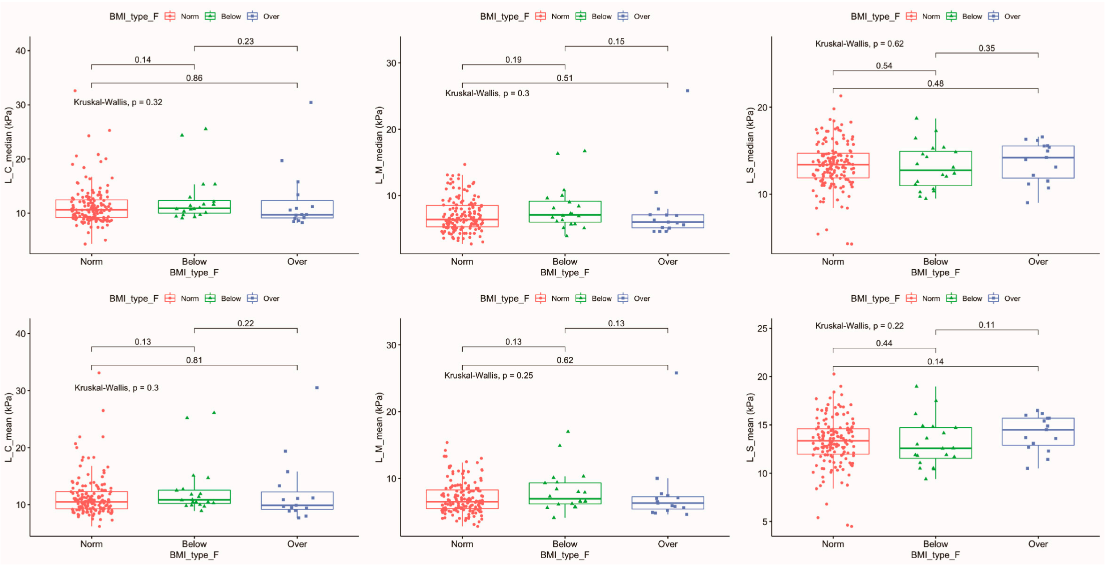

Supplementary Figure S3. Patients' serum creatinine and eGFR at baseline and last follow-up.

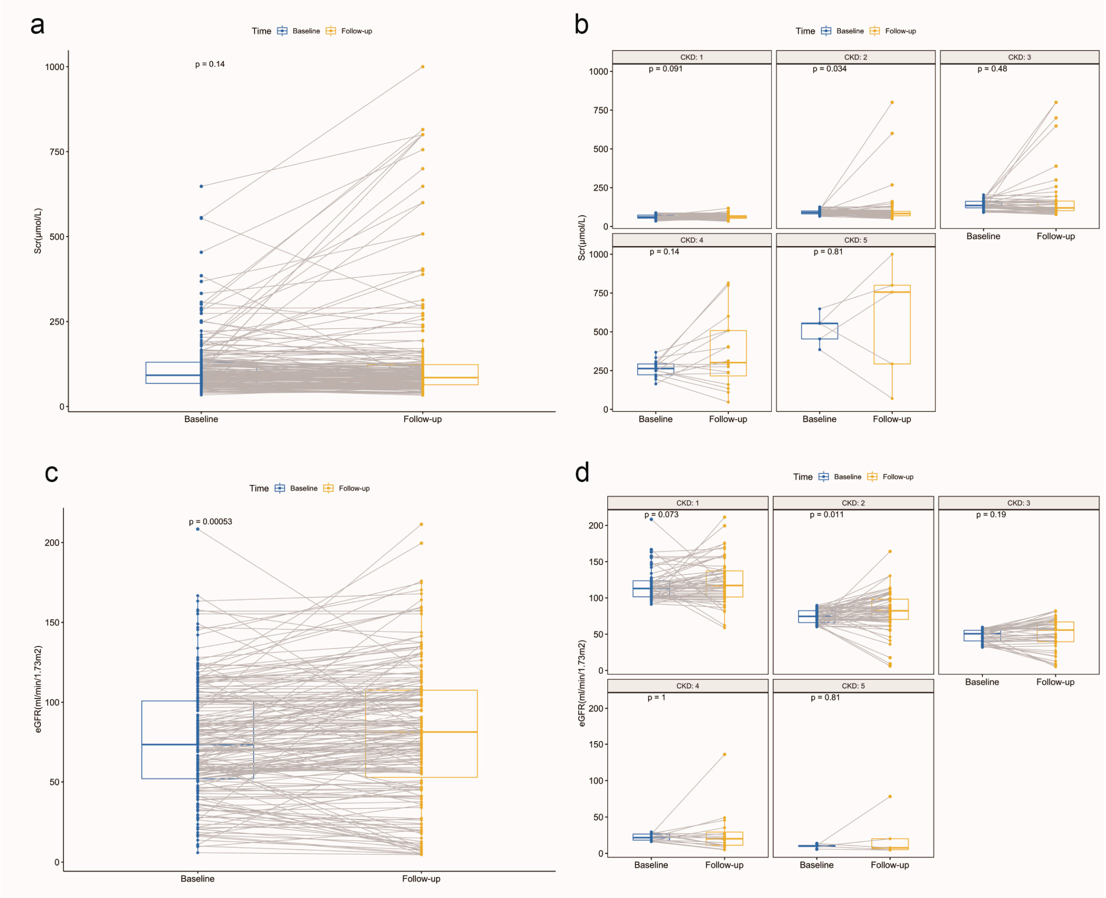

Supplementary Figure S4. Cutoff value based on Kaplan-Meier method

eGFR, eGFR at baseline; Scr, serum creatinine at baseline; ACR, urinary albumin to creatinine ratio at baseline; L_C_median, median SWE value of left renal cortex; L_S_mean, mean SWE value of left renal sinus; Length, length of left kidney.

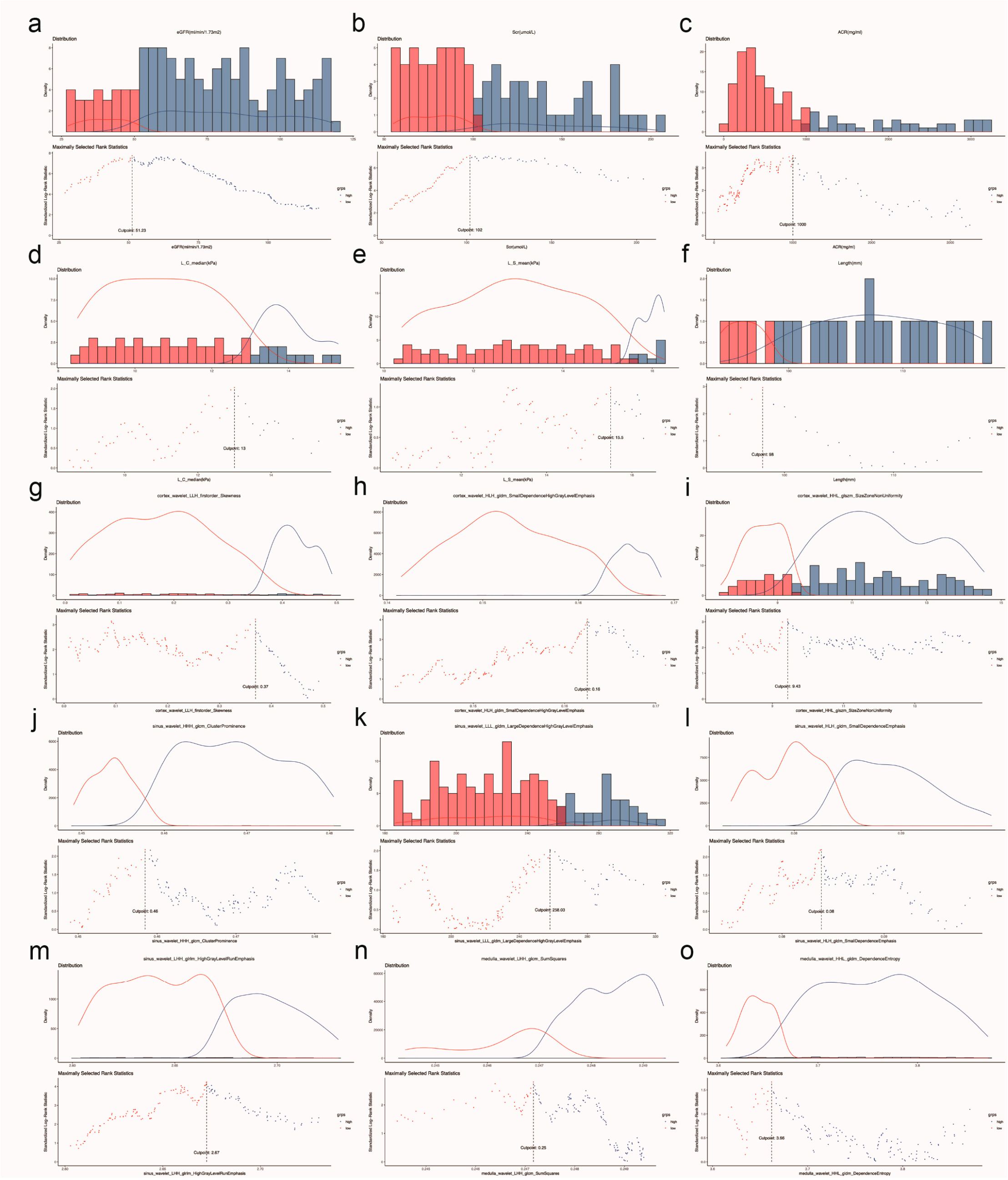

Supplementary Table S1. Test of proportional hazards assumption for the multivariate Cox regression model.

	Chi-square	Pvalue
eGFR(ml/min/1.73m^2)	1.680	0.195
Scr(μmol/L)	0.749	0.387
ACR(mg/g)	0.171	0.680
TA	4.190	0.242
A_C	5.410	0.144
L_C_median(kPa)	0.976	0.323
L_S_mean(kPa)	1.190	0.276
Length_mm(kPa)	0.956	0.328
cortex_wavelet_LLH_firstorder_Skewness	5.940	0.015
cortex_wavelet_HLH_gldm_SmallDependenceHighGrayLevelEmphasis	2.040	0.154
cortex_wavelet_HHL_glszm_SizeZoneNonUniformity	0.716	0.397
sinus_wavelet_LHH_glrlm_HighGrayLevelRunEmphasis	0.008	0.929
sinus_wavelet_HLH_gldm_SmallDependenceEmphasis	1.560	0.211
sinus_wavelet_HHH_glcm_ClusterProminence	0.022	0.881
sinus_wavelet_LLL_gldm_LargeDependenceHighGrayLevelEmphasis	0.000	0.998
medulla_wavelet_LHH_glcm_SumSquares	0.000	0.985
medulla_wavelet_HHL_gldm_DependenceEntropy	1.040	0.308
GLOBAL	28.900	0.117

eGFR, eGFR at baseline; Scr, serum creatinine at baseline; ACR, urinary albumin to creatinine ratio at baseline; TA, tubular atrophy; A_C, artery/arteriole hyalinosis. L_C_mean, mean SWE value of left renal cortex; L_C_median, median SWE value of left renal cortex; L_M_mean, mean SWE value of left renal medulla; L_M_median, median SWE value of left renal medulla; L_S_mean, mean SWE value of left renal sinus; L_S_median, median SWE value of left renal sinus; Length, length of left kidney.

Supplementary Table S2. The multicollinearity diagnosis for the multivariate Cox regression.

Parameters in multivariate regression	Tolerance	Variance inflation factor
eGFR(ml/min/1.73m2)	0.331	3.025
Scr(μmol/L)	0.375	2.666
ACR(mg/g)	0.949	1.053
TA	0.484	2.066
A_C	0.715	1.399
L_C_median(kPa)	0.943	1.060
Length(mm)	0.778	1.285
cortex_wavelet_LLH_firstorder_Skewness	0.822	1.216
cortex_wavelet_HLH_gldm_SmallDependenceHighGrayLevelEmphasis	0.924	1.082
cortex_wavelet_HHL_glszm_SizeZoneNonUniformity	0.843	1.186
sinus_wavelet_LHH_glrlm_HighGrayLevelRunEmphasis	0.856	1.169
sinus_wavelet_HLH_gldm_SmallDependenceEmphasis	0.789	1.268
sinus_wavelet_HHH_glcm_ClusterProminence	0.783	1.277
sinus_wavelet_LLL_gldm_LargeDependenceHighGrayLevelEmphasis	0.854	1.171
medulla_wavelet_LHH_glcm_SumSquares	0.912	1.096
medulla_wavelet_HHL_gldm_DependenceEntropy	0.870	1.149

eGFR, eGFR at baseline; Scr, serum creatinine at baseline; ACR, urinary albumin to creatinine ratio at baseline; TA, tubular atrophy; A_C, artery/arteriole hyalinosis. L_C_mean, mean SWE value of left renal cortex; L_C_median, median SWE value of left renal cortex; L_M_mean, mean SWE value of left renal medulla; L_M_median, median SWE value of left renal medulla; L_S_mean, mean SWE value of left renal sinus; L_S_median, median SWE value of left renal sinus; Length, length of left kidney.

Supplementary Table S3. C-index of Cox regression models.

Cox Regression Model	C-index(95%CI)
Model-All	0.9051(0.8460–0.9196)
Model-Clin+Patho	0.8540(0.7984–0.8920)
Model-Clin+SWE	0.8341(0.7727–0.8771)
Model-Clin+SWE+Radiomics	0.8724 (0.8105–0.8908)

Clin, clinical features of eGFR at baseline, Scr at baseline, ACR at baseline; Patho, pathological features of tubular atrophy, artery/arteriole hyalinosis; SWE, elastography parameters of median SWE value of left renal cortex, mean SWE value of left renal sinus; Radiomics, Radiomics signatures of cortex wavelet LLH firstorder Skewness, cortex wavelet HLH gldm SmallDependenceHighGrayLevelEmphasis, cortex wavelet HHL glszm SizeZoneNonUniformity, sinus wavelet LHH glrlm HighGrayLevelRunEmphasis, sinus wavelet HLH gldm SmallDependenceEmphasis, sinus wavelet HHH glcm ClusterProminence, sinus wavelet LLL gldm LargeDependenceHighGrayLevelEmphasis, medulla wavelet LHH glcm SumSquares, medulla wavelet HHL gldm DependenceEntropy; Model1-All, Cox regression model of all features; Model2-Clin+Patho, Cox regression model of clinical and pathological features and length of left kidney; Model3-Clin+SWE, Cox regression model of clinical features, length of left kidney, elastography parameters; Model4-Clin+SWE+Radiomics, Cox regression model of clinical features, length of left kidney, elastography parameters, and radiomics signatures.

Supplementary Table S4. Comparison of time-–dependent ROCs of Cox regression models

Cox Regression Model	t=12	t=24	t=30
Model-Clin+Patho vs Model-Clin+SWE	0.9045	0.1084	0.8531
Model-Clin+Patho vs Model-Clin+SWE+Radiomics	0.4927	0.6925	0.0099
Model-Clin+Patho vs Model-All	0.9997	0.9547	0.0024
Model-Clin+SWE+Radiomics vs Model-All	0.4927	0.6925	0.0099

The areas under curves (AUCs) at 12, 24, 30 months of time-dependent ROCs were calculated and compared using the method of Hanley and McNeil for a single time point; Data in the sheet are adjusted p value; t, time; Clin, clinical features of eGFR at baseline, Scr at baseline, ACR at baseline; Patho, pathological features of tubular atrophy, artery/arteriole hyalinosis; SWE, elastography parameters of median SWE value of left renal cortex, mean SWE value of left renal sinus; Radiomics, Radiomics signatures of cortex wavelet LLH firstorder Skewness, cortex wavelet HLH gldm SmallDependenceHighGrayLevelEmphasis, cortex wavelet HHL glszm SizeZoneNonUniformity, sinus wavelet LHH glrlm HighGrayLevelRunEmphasis, sinus wavelet HLH gldm SmallDependenceEmphasis, sinus wavelet HHH glcm ClusterProminence, sinus wavelet LLL gldm LargeDependenceHighGrayLevelEmphasis, medulla wavelet LHH glcm SumSquares, medulla wavelet HHL gldm DependenceEntropy; Model1-–All, Cox regression model of all features; Model2-–Clin+Patho, Cox regression model of clinical and pathological features and length of left kidney; Model3-–Clin+SWE, Cox regression model of clinical features, length of left kidney, elastography parameters; Model4-–Clin+SWE+Radiomics, Cox regression model of clinical features, length of left kidney, elastography parameters, and radiomics signatures.

Supplementary Table S5. Baseline characters of the train and test cohort

	Total (n=187)	Train(n=149)	Test(n=38)	P-value
Age(year)	45.00(32.00–59.00)	46.00(32.00–59.00)	44.00(35.00–54.00)	0.846
Sex(male%)	105(56.1%)	88(83.80%)	17(16.20%)	0.112
BMI(kg/m^2)	24.30(21.96–27.16)	24.57(22.05–27.39)	23.14(21.06–25.66)	0.111
SBP(mmHg)	141.00(121.00–165.00)	146.00(120.50–166.00)	141.00(123.75–163.75)	0.732
DBP(mmHg)	77.50(70.00–85.75)	78.00(69.00–85.00)	76.50(69.25–88.75)	0.797
eGFR(MDRD)(ml.min/1.73m^2)	73.35(51.96–101.88)	75.07(52.84–101.63)	61.82(42.71–100.59)	0.197
Scr(μmol/L)	92.00(68.00–131.00)	89.00(68.00–128.00)	96.50(64.75–165.50)	0.419
BUN(mmol/L)	5.50(4.23–7.38)	5.50(4.30–7.25)	5.35(4.10–7.65)	0.838
UA(μmol/L)	363.50(299.50–412.50)	363.00(300.00–402.50)	402.00(302.75–440.25)	0.161
Alb(g/L)	35.60(29.95–41.05)	35.40(29.35–40.65)	36.90(29.51–41.30)	0.577
24hUpro(mg)	1454.40(624.60–3397.15)	1524.60(646.50–3544.50)	1304.85(551.40–2466.75)	0.249
ACR(mg/g)	565.95(253.03–1788.85)	568.50(264.65–1751.20)	543.70(241.85–1084.50)	0.501

**Pathological Changes**	**Total (n=187)**	**Train(n=149)**	**Test(n=38)**	**P-value**
Glomerular_Global Sclerosis	20.00%(5.88%–43.75%)	20.00%(5.38%–42.26%)	20.00%(4.55%–50.00%)	0.656
Glomerular_Focal Segmental Sclerosis	0.00%(0.00%–7.69%)	0.00%(0.00%–7.55%)	0.00%(0.00%–8.46%)	0.871
Glomerular_Crescents	0.00%(0.00%–3.33%)	0.00%(0.00%–0.00%)	0.00%(0.00%–5.05%)	0.386
Glomerular_Fibrinoid necrosis	0.00%(0.00%–0.00%)	0.00%(0.00%–0.00%)	0.00%(0.00%–0.00%)	0.403
Mesengial Matrix hyperplasia				0.790
0	17(9.10%)	13(76.50%)	4(23.50%)	
1	140(74.90%)	112(80.00%)	28(20.00%)	
2	20(10.70%)	17(85.00%)	3(15.00%)	
3	10(5.30%)	7(70.00%)	3(30.00%)	
Mesangial hypercellularity				0.254
0	30(16.00%)	24(80.00%)	6(20.00%)	

1	134(71.70%)	108(80.60%)	26(19.40%)	
2	22(11.80%)	17(77.30%)	5(22.70%)	
3	1(0.50%)	0(0.00%)	1(100.00%)	
Intra-capillary proliferation				0.748
0	144(77.00%)	115(79.90%)	29(20.10%)	
1	2(1.10%)	2(100.00%)	0(0.00%)	
2	41(21.90%)	32(78.00%)	9(22.00%)	
3	0(0.00%)	0(0.00%)	0(0.00%)	
Capillary wall hyalinosis				0.094
0	114(61.00%)	85(74.60%)	29(25.40%)	
1	49(26.20%)	42(85.70%)	7(14.30%)	
2	21(11.20%)	20(95.20%)	1(4.80%)	
3	3(1.60%)	2(66.70%)	1(33.30%)	
Tubular atrophy				0.526
0	14(7.50%)	12(85.70%)	2(14.30%)	
1	88(47.10%)	71(80.70%)	17(19.30%)	
2	63(33.70%)	51(81.00%)	12(19.00%)	
3	22(11.80%)	15(68.20%)	7(31.80%)	
Interstitial inflammation				0.536
0	14(7.50%)	13(92.90%)	1(7.10%)	
1	91(48.70%)	72(79.10%)	19(20.90%)	
2	60(32.10%)	48(80.00%)	12(20.00%)	
3	22(11.80%)	16(72.70%)	6(27.30%)	
Interstitial fibrosis				0.480
0	13(7.00%)	12(92.30%)	1(7.70%)	

1	92(49.20%)	72(78.30%)	20(21.70%)	
2	61(32.60%)	50(82.00%)	11(18.00%)	
3	21(11.20%)	15(71.40%)	6(28.60%)	
Artery/arteriole intima thickening				0.396
0	79(42.20%)	64(81.00%)	15(19.00%)	
1	39(20.90%)	34(87.20%)	5(12.80%)	
2	59(31.60%)	44(74.60%)	15(25.40%)	
3	10(5.30%)	7(70.00%)	3(30.00%)	
Artery/arteriole hyalinosis				0.249
0	105(56.10%)	80(76.20%)	25(23.80%)	
1	47(25.10%)	42(89.40%)	5(10.60%)	
2	21(11.20%)	17(81.00%)	4(19.00%)	
3	14(7.50%)	10(71.40%)	4(28.60%)	
Chronic change				0.519
Minical chronic changes(0-1)	10(5.30%)	9(90.00%)	1(10.00%)	
Mild chronic changes(2-4)	66(35.30%)	52(78.80%)	14(21.20%)	
Moderate chronic changes(5-7)	61(32.60%)	51(83.60%)	10(16.40%)	
Severe chronic changes(≥8)	50(26.70%)	37(74.00%)	13(26.00%)	

**Ultrasound Parameters**	**Total (n=187)**	**Train(n=149)**	**Test(n=38)**	**P-value**
Mean SWE value of left renal cortex (kPa)	10.60(9.50–12.50)	10.50(9.30–12.40)	10.55(9.68–12.50)	0.606
Median SWE value of left renal cortex (kPa)	10.60(9.20–12.60)	10.60(9.15–12.60)	10.65(9.45–12.45)	0.618
Mean SWE value of left renal medulla (kPa)	6.50(5.50–8.20)	6.50(5.50–8.10)	6.50(5.68–9.00)	0.586
Median SWE value of left renal medulla (kPa)	6.50(5.30–8.40)	6.40(5.20–8.30)	6.60(5.68–9.23)	0.365
Mean SWE value of left renal sinus (kPa)	13.40(12.10–14.80)	13.40(12.05–14.80)	13.20(11.70–14.95)	0.764
Median SWE value of left renal sinus (kPa)	13.40(11.90–14.90)	13.40(11.80–14.80)	13.30(11.58–15.70)	0.928

Length_left kidney (mm)	106.00(99.00–112.00)	106.00(98.00–112.50)	105.50(99.75–110.00)	0.945
Width_left kidney (mm)	45.00(42.00–49.00)	45.00(42.00–49.00)	44.00(41.00–48.00)	0.415
Thickness_left kidney (mm)	43.40(39.00–46.50)	44.00(40.00–47.00)	42.00(38.75–45.00)	0.069
Kidney volume(cm^3)	201.35(173.04–238.66)	206.19(175.37–242.82)	188.27(168.05–230.97)	0.279

CKD fo_1~2, patients at CKD stage 1~2 at the last follow–up time. CKD fo_3~5, patients at CKD stage 3~5 at the last follow-up time; G_G_Sclerosis, Glomerular_Global Sclerosis; G_FS_Sclerosis, Glomerular_Focal Segmental Sclerosis; G_Crescents, Glomerular_Crescents; G_Fibrinoid necrosis, Glomerular_Fibrinoid necrosis; L_C_mean, mean SWE value of left renal cortex; L_C_median, median SWE value of left renal cortex; L_M_mean, mean SWE value of left renal medulla; L_M_median, median SWE value of left renal medulla; L_S_mean, mean SWE value of left renal sinus; L_S_median, median SWE value of left renal sinus; Length,Width, Thickness, and Kidney volume, are length, width, thickness, and product of length and width and thickness of left kidney respectively. All parameters were collected at the time of biopsy.
